# Influence of Anaemia on Multifactorial Disease Retinopathy of Prematurity: A Prospective Observational Study

**DOI:** 10.7759/cureus.27877

**Published:** 2022-08-11

**Authors:** Manish Tandon, Raksha Ranjan, Uma Muralidharan, A Kannan

**Affiliations:** 1 Ophthalmology, United Institute of Medical Sciences, Prayagraj, IND; 2 Pediatrics and Neonatology, Adesh Institute of Medical Sciences and Research, Bathinda, IND; 3 Pediatrics and Child Health, Meenakshi Mission Hospital and Research Centre, Madurai, IND

**Keywords:** screening, blindness, preterm, anaemia, retinopathy of prematurity

## Abstract

Background: Retinopathy of prematurity (ROP), a preventable cause of childhood blindness, is a severe complication of preterm (PT) birth treatment.

Purpose: The purpose of this study is to analyse the risk factors (RF) associated with the development and progression of ROP. Particular focus is on the contribution of anaemia towards the development and progression of ROP.

Methods: This study is a prospective observational study done in the Department of Paediatrics at Meenakshi Mission Hospital & Research Centre, Madurai, over 12 months from May 2013 to April 2014. The study included all consecutively admitted neonates born in and out of the hospital with gestational age (GA) less than or equal to 35 weeks or birth weight (BW) less than or equal to 2 kg and assessed for the gestational, perinatal, and postnatal RF. In addition, at the time of ROP screening, haemoglobin (Hb) and haematocrit (Hct) were checked. The statistical analysis was performed by Stata 11.1 (StataCorp LLC, College Station, TX).

Result: The incidence of ROP in our study (46.7%) is higher than previously reported in India. In our study, GA and weight of the neonate at birth have a significant association with ROP incidence. Anaemia in our study is significantly associated with ROP incidence but not as an independent RF. The outcome of various stages of ROP is statistically significant, showing early stages 1 and 2 have more chances of spontaneous regression, and stages 3 and 4 are more likely to need treatment. Two cases in our study with stage 4 ROP had no complications, and none had stage 5 disease.

Conclusion: Anaemia should be avoided or corrected in PT newborns as it is a potential and avoidable RF for ROP development. The limitation of our study is the small sample size, and probably more extensive randomized trials will help make this association clear. We recommend ROP screening for PT babies with GA less than 35 weeks and BW less than 2 kg who have the RF amounting to screening and done as per protocol.

## Introduction

Retinopathy of prematurity (ROP) is a significant cause of preventable blindness in children worldwide [1]. As a result of improved neonatal care, preterm (PT) neonates' survival has increased, alongside an increase in morbidities like ROP. Middle-income countries like India face the third ROP epidemic [2]. Due to ROP, India accounts for nearly 10% of blindness and visual impairment worldwide [3]. ROP represents a multifactorial disease, and now it is well recognized that early gestational age (GA) ≤ 30 weeks and low birth weight (BW) ≤ 1.5 kg are the crucial risk factors (RF) in the development of ROP along with oxygen therapy. The other factors that have a significant impact on ROP are poor weight gain, percentage of oxygen in the inhaled air, hypoxia, respiratory distress syndrome (RDS), anaemia, blood transfusion (BT), and sepsis [3]. In addition, reported are intraventricular haemorrhage (IVH), apnoea, hypercarbia or hypocarbia, patent ductus arteriosus (PDA), bronchopulmonary dysplasia (BPD), and perinatal asphyxia to affect the occurrence of ROP [4,5].

The exact pathogenesis of ROP is still unknown. The first observation in the acute phase is the cessation of vasculogenesis. Later in the disease, peripheral hypoxia develops and produces vascular endothelial growth factors (VEGFs) in the non-vascularized retina. These growth factors stimulate abnormal vasculogenesis, and neovascularization can occur. Because of poor pulmonary function, a state of relative retinal hypoxia occurs, which causes upregulation of VEGF, which, in the susceptible neonate, can cause abnormal fibrovascular growth. This neo-vascularization can then lead to scarring and vision loss.

The central purpose of any ROP screening program is the early identification and prompt treatment of threshold (T)-ROP. Hence, identifying RF predisposing PT neonates for ROP development would have important therapeutic implications. A lot of babies who are sick show worsening of ROP, and a simple test such as a haemoglobin check and correction of anaemia can not only improve the general health of the baby but also very often will save the unnecessary treatment of ROP by laser or intravitreal injection. This simple procedure is the novelty of our approach towards treating ROP and looking at it as part of systemic disease worsening and not eyes alone.

## Materials and methods

This study is a prospective observational study done in the neonatal intensive care unit (NICU) of the Department of Paediatrics, Meenakshi Mission Hospital & Research Centre, Madurai, a tertiary care facility in south-central India, with the approval of the ethical and scientific board of the hospital, over 12 months from May 2013 to April 2014. The babies meeting the inclusion criteria admitted consecutively to the NICU, born in and out of the hospital, were enrolled in the study after obtaining informed consent from the parents and attendants of the respective babies. Table 1 shows the inclusion and exclusion criteria of the study to enrol participants.

**Table 1:** Inclusion and exclusion criteria

Inclusion criteria	Exclusion criteria
All preterm neonates weighing less than or equal to 2 kg or gestational age less than or equal to 35 weeks at birth [6,7]	All infants more than 35 weeks of gestational age and more than 2 kg at birth
	Patients with major congenital malformations, chromosomal abnormalities, and inborn errors of metabolism
	For whom parents did not give consent for the study
	Babies who died or got discharged against medical advice
	Babies who were lost to follow up

The ophthalmologist performed the retinal examinations in the NICU under the supervision of the attending paediatrician. Babies received adequate ventilatory support during the procedure with cardio-respiratory monitoring and sedation were avoided. Anterior segment examination and pupillary reactions were noted first. Then, pupils were dilated with tropicamide 0.5% and phenylephrine 2.5%, instilled twice at five-minute intervals in both eyes.

The examination was performed with an indirect ophthalmoscope and a +20-dioptre lens. The findings (class, stage, diagrams) were recorded according to the International Classification of ROP-2 (ICROP-2) [8]. Follow-up examinations were done according to retinal findings until the outcome of either ROP regressed without treatment or ROP was treated.

Patients were treated according to the ROP stage and severity of disease, divided into type 1 (high-risk pre-T-ROP) and type 2 (lower risk pre-T-ROP) as per early treatment of ROP (ETROP) guidelines [9]. Type 1 ROP refers to zone I, any stage ROP with plus disease; zone I, stage 3 ROP without plus disease; or zone II, stage 2 or 3 ROP with plus disease. Type 2 ROP refers to zone I, stage 1 or 2 ROP without plus disease; or zone II, stage 3 ROP without the plus disease [8].

The haemoglobin (Hb) and haematocrit (Hct) were checked at ROP screening and as and when required. We defined anaemia as Hb value < 10 gm/dl or Hct < 30% and severe anaemia as Hb < 8 gm/dl or Hct < 24% and were derived from transfusion thresholds suggested for newborns [10].

Statistical methods

The analysis was performed by Stata 11.1 (StataCorp LLC, College Station, TX). The continuous variables were expressed as mean and standard deviation (SD). Categorical variables were expressed as frequency and percentage. An independent t-test was used to determine the significant difference between groups (ROP present and absent) and RF. The chi-square test and Fisher's exact test were used to determine the association between the categorical variables. Logistic regression was used to find the odds ratio (OR) between the dependent variable "ROP" and independent variables, including GA, BW, anaemia, BT, oxygen, mechanical ventilation (MV), and continuous positive pressure ventilation (CPAP). P < 0.05 was considered statistically significant.

## Results

During the study period, 105 babies fulfilled the inclusion criteria out of 137 babies screened and were eligible for the study. At the end of one year, 59 infants, 28 (47.5.%) males and 31 (52.54%) females, completed the study, and their data were analysed. Data of 46 babies were excluded from the study analysis; 10 patients were excluded due to congenital anomalies, chromosomal abnormalities, and inborn errors of metabolism, 26 babies were excluded due to death or discharge against medical advice, and 10 babies were lost to follow up.

The mean BW and GA for the babies in the present study are 1.63 ± 0.27 kg and 32.92 ± 1.57 weeks., respectively. The mean Hb value of the whole sample with ROP is 9.98 ± 3.94 g/dl, and without ROP is 12.15 ± 4.82 g/dl.

Among 59 infants, 49 developed ROP; 23 (46.94%) were males and 26 (53.06%) were females. Both the eyes showed similar findings of ROP. Stagewise cumulative incidence of ROP is shown in Table 2: stage 1 (14, 23.7%), stage 2 (11, 18.6%), stage 3 (22, 37.2%), and stage 4 (2, 3.4%).

**Table 2:** Incidence of ROP in the study group ROP: retinopathy of prematurity.

ROP stage	N (%)
Mature retina	10 (16.95)
Stage 1 ROP	14 (23.7)
Stage 2 ROP	11 (18.6)
Stage 3 ROP	22 (37.2)
Stage 4 ROP	2 (3.4)

Two cases in our study with stage 4 ROP had no complications, and none had stage 5 disease. Plus disease was seen in 18 neonates, with stage 1 ROP in four and stage 3 ROP in 14.

Out of 49 patients developing ROP, 38 had Hb values < 10 g/dl. Among these 38 cases with anaemia, the mean Hb value was 8.11 (4.8-9.9), median = 8.7 and mode = 9.5, with an SD of 1.46. The mean Hb value of the whole sample with ROP was 9.98 ± 3.94 g/dl and without ROP was 12.15 ± 4.82 g/dl.

The study group's univariate analysis (Table 3) of RF with ROP incidence shows GA, BW, and anaemia correlate significantly with ROP incidence. However, with logistic regression analysis (Table 4), GA is the independent RF for ROP development in our study. In contrast, anaemia is the potential RF for ROP development only in the presence of other factors; the same is found true for BW, oxygen therapy, MV, and CPAP. Thus, anaemia is significantly associated with the ROP incidence but not as an independent RF in this study.

**Table 3:** Univariate analysis of the incidence of ROP in the study group ROP: retinopathy of prematurity; BW: birth weight; GA: gestational age; BPD: bronchopulmonary dysplasia; RDS: respiratory distress syndrome; HMD: hyaline membrane disease; FIO2: fraction of inspired oxygen; CPAP: continuous positive pressure ventilation; PIH: pregnancy-induced hypertension; NVD: normal vaginal delivery; LSCS: lower segment caesarean section.

	Non-ROP	ROP	P-value
No. of patients	10	49	
BW, mean (SD)	1.59 (0.32)	1.64 (0.27)	0.043
GA, mean (SD)	33.2 (1.40)	32.86 (1.59)	0.033
Sex			1
Male	5 (8.47)	23 (46.94)	
Female	5 (8.47)	26 (53.06)	
Fetal distress	3 (5.1)	21 (35.6)	0.506
RDS	9 (15.3)	47 (79.7)	0.433
HMD	5 (8.5)	19 (32.2)	0.725
Surfactant therapy	5 (8.5)	17 (28.8)	0.477
Apnoea	2 (3.4)	15 (25.4)	0.708
Hyperbilirubinemia	6 (10.2)	37 (62.7)	0.436
Phototherapy	6 (10.2)	37 (62.7)	0.436
Sepsis	4 (6.8)	19 (32.23)	0.589
BPD	1 (1.7)	10 (16.9)	0.67
Oxygen therapy	9 (15.25)	48 (81.4)	0.313
FIO2			0.722
24-44%	3 (5.1)	21 (35.6)	
44-100%	6 (10.3)	28 (47.5)	
Mechanical ventilation	6 (10.17)	29 (49.15)	1
CPAP	6 (10.17)	34 (57.63)	0.712
Anaemia	4 (6.78)	38 (64.4)	0.027
No anaemia	6 (10.2)	11 (18.6)	
Blood transfusion	3 (5.08)	31 (52.5)	0.8
Antenatal steroids	6 (10.2)	22 (37.3)	0.494
PIH	2 (3.4)	14 (23.7)	0.713
Gestational diabetes in mother	1 (1.7)	2 (3.4)	0.433
Mode of delivery			0.259
NVD	9 (15.3)	34 (57.6)	
LSCS	1 (1.7)	15 (25.4)	

**Table 4:** Logistic regression analysis of risk factors of ROP CPAP: continuous positive airway pressure; ROP: retinopathy of prematurity.

Potential risk factor	Odds ratio	P-value	95% CI
Gestational age	0.528	0.013	0.319-0.872
Birth weight	0.181	0.122	0.021-1.579
Anaemia	0.892	0.138	0.767-1.037
Blood transfusions	4.01	0.06	0.92-17.51
Oxygen	5.33	0.252	0.305-93.29
Mechanical ventilation	1.05	0.942	0.26-4.22
CPAP	1.67	0.477	0.407-6.82

BT is not a significant RF for ROP as per the present study. Other factors like gender, mode of delivery, fetal distress, APGAR (appearance, pulse, grimace, activity, and respiration) score, multiple births, hyperbilirubinemia, RDS, hyaline membrane disease (HMD), surfactant, hypoxic-ischemic encephalopathy (HIE), apnoea, PDA, BPD, sepsis, IVH, maternal factors like pregnancy-induced hypertension (PIH), gestational diabetes mellitus (GDM), antepartum haemorrhage, antenatal steroid intake, and prolonged rupture of membranes have not shown statistically significant association with the development of ROP.

Analysis of each stage for its outcome, as spontaneous regression of the ROP group and the group requiring any mode of treatment, is presented in Table 5. In 23 (46.94%) patients, ROP regressed spontaneously, and 26 (53.06%) patients underwent treatment for ROP. In stage 1, out of 14 (28.56%), four neonates who had plus disease ROP received treatment, and in the remaining 10 (20.4%), ROP regressed spontaneously, whereas, in stage 3, out of 22 (44.86%), 18 (36.7%) needed treatment. The outcome of each stage of ROP is statistically significant. The outcome of ROP in babies with anaemia has no significant association with whether needing treatment or spontaneous regression of ROP.

**Table 5:** Analysis of stagewise outcome of ROP ROP: retinopathy of prematurity.

	Regressed ROP	Treated ROP	P-value
No. of patients	23	26	
Stage 1	10 (20.4)	4 (8.16)	<0.001
Stage 2	9 (18.36)	2 (4.08)	
Stage 3	4 (8.16)	18 (36.7)	
Stage 4	0	2 (4.08)	
Anaemia	16 (32.6)	22 (44.9)	0.424

The maximum number of babies developing ROP was 38 (BW < 1.5 kg), but 11 of 17 babies in the BW range of 1.5-2 kg also developed ROP. GA of 30-32 weeks (n = 33) had the maximum number of ROP (n = 29) as compared to other groups, i.e., <30 weeks (n = 10), where all developed ROP, and 32-35 weeks (n = 16), out of which 10 developed ROP, which was statistically significant (Table 6).

**Table 6:** Association between ROP and independent variables (gestational age and birth weight) ROP: retinopathy of prematurity.

Weight in kilogram (kg)	ROP present		ROP absent		P-value
	f	%	f	%	
1-1.25	18	30.5	2	3.4	0.043
1.25-1.5	20	40.8	2	3.4	
1.5-1.75	2	3.4	3	5.1	
1.75-2	9	18.6	3	5.1	
Total	49	83.1	10	16.95	
Gestational age (in weeks)					
<30	10	16.9	0	0	0.033
30-32	29	49.2	4	6.8	
33-35	10	16.9	6	10.2	
Total	49	83.05	10	16.9	

ROP regressed spontaneously in 23 (46.94%) neonates, and the rest of the babies (26) in the cohort required either intraocular bevacizumab, photocoagulation, or both as treatment of ROP (Table 7).

**Table 7:** Outcome of ROP ROP: retinopathy of prematurity.

Outcome	ROP present	
	f	%
Regressed without treatment	23	46.94
Intraocular injection (IOI)	6	12.24
Photocoagulation (PHC)	15	30.61
Both (IOI and PHC)	5	10.20
Total	49	83.05

## Discussion

In our study, the incidence of ROP is 46.7%, which is higher than the previously reported incidence from studies in India of 24% [11] and comparable to the incidence previously reported in other studies in India [7,12-15]. The higher incidence can be explained as a result of improved neonatal survival, supplemental oxygen therapy, etc., due to the availability of better intensive care services for sick newborns.

A total of 23 males and 26 females developed ROP. The incidence of ROP is independent of whether the neonate is male or female in the present study. Many other studies also noted a similar observation [16-18]. However, the male sex was a significant risk factor for developing severe ROP compared with females in some studies [19,20]. ROP is a multifactorial disease including low GA, low BW, sepsis, oxygen therapy, RDS, and BT known to influence ROP incidence [21]. However, many studies show that the most significant RF for ROP development were low GA and low BW [22-26].

The mean GA for the babies in the present study was 32.92 ± 1.57 weeks, which is higher than 29.7 weeks [27] and 30.3 weeks [28,29], and reflects the variation in sample composition of these studies. Our study documents a statistically significant association between ROP and GA, agreeing with the results of studies done by Shah et al. [27], Fortes et al. [30], and others [29,31].

In this study, a maximum number of ROP cases were in the age group 30-33 weeks; this is because a proportionately more number of babies belonged to this age group, but the proportion of babies who developed ROP was more in the <30 weeks group, as all the babies falling in the group developed ROP.

The present study agrees with many studies [23,24,27,30], which reported that lower BW was significantly associated with ROP development and explains the increased susceptibility to oxygen therapy, prolonged ventilation, sepsis, and BT in very low BW infants. Maximum babies, 36.19% developing ROP in this study, are in the low BW range (1-1.25 kg and 1.25-1.5 kg), comparable to that reported in other studies done among the very low BW infants [18,26]. A fact not to be ignored is that 11 of 17 babies in the BW range of 1.5-2 kg also developed ROP. Bigger and more mature babies developing ROP are usually sicker and more prone to comorbidities due to varying care standards [32,33].

Our study found that anaemia is a significant RF for ROP development, and a significant association was observed in various other studies [14,34,35]. The relationship between anaemia to ROP is challenging due to many factors affecting blood Hb levels, especially BT. In addition, the decision to transfuse blood is affected by other variables, such as lung disease, oxygen status, and the infant's overall health. As ROP is a multifactorial disease, anaemia may have a significant role, knowing that VEGF drives it. Hence, if the VEGF is more due to less oxygen delivery, the correction of anaemia should improve oxygen delivery and lessen VEGF levels in the eye, thus regressing ROP. Bossi et al. [11] evaluated the association between anaemia and ROP in infants weighing less than 1.5 kg and found no association. When considering Hb levels during the first week of life, Alter et al. [36] found no difference between infants with < stage 2 ROP and infants with > stage 3 ROP. Brooks et al. [37] found no association between anaemia or BT and ROP incidence or severity. In disagreement with Chawla et al., our study does not show the association between BT and ROP [38].

Mean Hb values for each stage of ROP in our study group are as follows: stage 1 (10.41 ± 4.88 g/dl), stage 2 (10.56 ± 4.16 g/dl), stage 3 (9.47 ± 3.43 g/dl), stage 4 (9.3 ± 0.848 g/dl), and matured retina (12.15 ± 4.83 g/dl). The outcome of each stage of ROP is statistically significant. The outcome of ROP in babies with anaemia has no significant association with whether needing treatment or spontaneous regression of ROP.

Mittelman and Cronin [39] found that infants weighing less than 1.36 kg at birth who developed ROP received more BT and oxygen therapy.

In agreement with various studies, we found an insignificant relationship between the mode of delivery and ROP occurrence [40,41]; nevertheless, this disagreed with others who found that caesarean section delivery was significantly associated with ROP occurrence [27,42].

In contrast to this study, many studies have found RDS, the number of blood units transfused, and the number of ventilated days to be significant RF for developing severe ROP requiring treatment among the screened population [5,32,38,43,44].

In disagreement with Shah et al. [27] and others [32,44], sepsis in this study is not significantly associated with ROP development. On the other hand, this agreed with Chaudhari et al. [45]. In agreement with other studies, in this study, oxygen therapy is an insignificant RF for ROP development [29,46], which disagreed with Darlow et al. [19].

We found that MV and CPAP were non-significant RF for ROP, which agreed with Murthy et al. [47]. However, others observed that MV and CPAP were significantly associated with ROP development [27,29,32,48]. The mean number of days of oxygen, MV, and CPAP between the ROP developed group and ROP not developed group is nearly identical with the insignificant p-value.

All ROP types and stages seen in this study were followed up till complete retinal vascularization. ROP regressed in six babies out of 11 cases who received anti-VEGF drug (bevacizumab), and the remaining five required photocoagulation. In zone I type 1 ROP, bevacizumab injection is an effective treatment; however, some cases may progress and require surgical management [49].

## Conclusions

Anaemia should be avoided or corrected in PT newborns as it is a potential and avoidable RF for ROP development. The limitation of our study is the small sample size and considerable patient attrition. Probably more extensive randomized trials will help make this association clear. Low BW and low GA are the most critical RF for ROP. We recommend ROP screening for PT babies with GA less than 35 weeks and BW less than 2 kg who have the RF amounting to screening and done as per protocol. Paediatrician-ophthalmologist coordination, superior NICU care practices, and effective ROP screening and treatment services can prevent ROP and reduce disease severity and morbidity. Routine screening is the best way to avoid severe stages of ROP and its complications.
